# A case report of atypical Kawasaki disease presented with severe elevated transaminases and literature review

**DOI:** 10.1186/s12879-021-06101-y

**Published:** 2021-05-04

**Authors:** Yifan Ren, Chuanxin Zhang, Xiaoqin Xu, Yu Yin

**Affiliations:** Department of Paediatrics, ShaoXing KeQiao Women And Children’s Hospital, ShaoXing, 312030 Zhejiang Province PR China

**Keywords:** Atypical Kawasaki disease, Kawasaki disease, Severely elevated transaminases, Children, Case report

## Abstract

**Background:**

Kawasaki disease (KD) is the most common cause of acquired heart disease among children in developed countries, in which the resulting coronary artery (CA) abnormalities cause myocardial ischemia, infarction, and death. Prompt diagnosis was essential, and supplemental information should be used to assist the diagnosis when classical clinical criteria are incomplete. The elevated levels of serum transaminases in most KD patients are mild. Herein, a case of atypical KD child with severely elevated transaminase was reported.

**Case presentation:**

A child with clinical manifestations of fever, high C-reactive protein (CRP) and severely elevated transaminases was reported. The treatment effect of antibiotic and liver-protecting drugs was not satisfactory. A bilateral diffuse dilation of the CA was detected on echocardiography on day 5 of the illness; thus, atypical KD was diagnosed. Elevated transaminases declined rapidly to normal after the treatment of intravenous immunoglobulin (IVIG). A 1-month follow-up revealed that CA returned to normal, and 2-month, 6-months, and 1-year follow-up revealed the child was in good general health.

**Conclusions:**

This case highlighted that atypical KD clinical symptoms were diverse, and severely elevated transaminases might provide a clue to healthcare providers for the diagnosis and management of atypical KD.

## Background

Kawasaki disease (KD) is an acute vasculitis, which commonly affects children between the age of 6 months and 5 years. Although the precise cause of the disease is yet unknown, a common pathway in many infectious or environmental factors that trigger inflammation of the blood vessels in individuals with a genetic predisposition to this disease could be ascribed as the putative factors [1]. Elevated serum liver enzymes constituted the typical laboratory tests manifested in the gastrointestinal tract of KD [2]. However, severely elevated transaminases could not be differentiated from atypical KD and infectious diseases, thereby delaying treatment.

## Case presentation

**Fig. 1:**
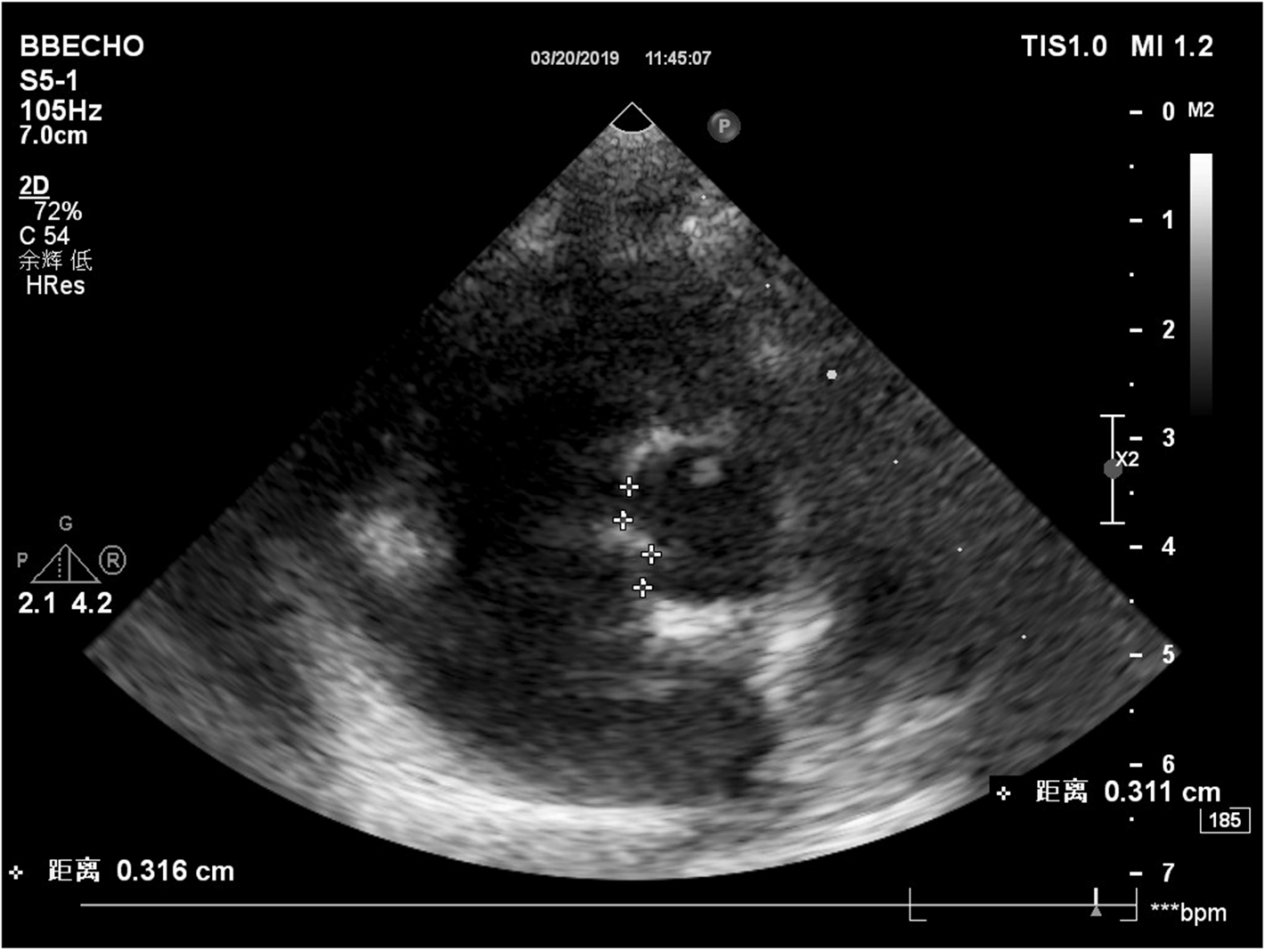
Right CA aneurysm (3.1 mm) was detected on echocardiography on day 5 of hospitalization

**Fig. 2:**
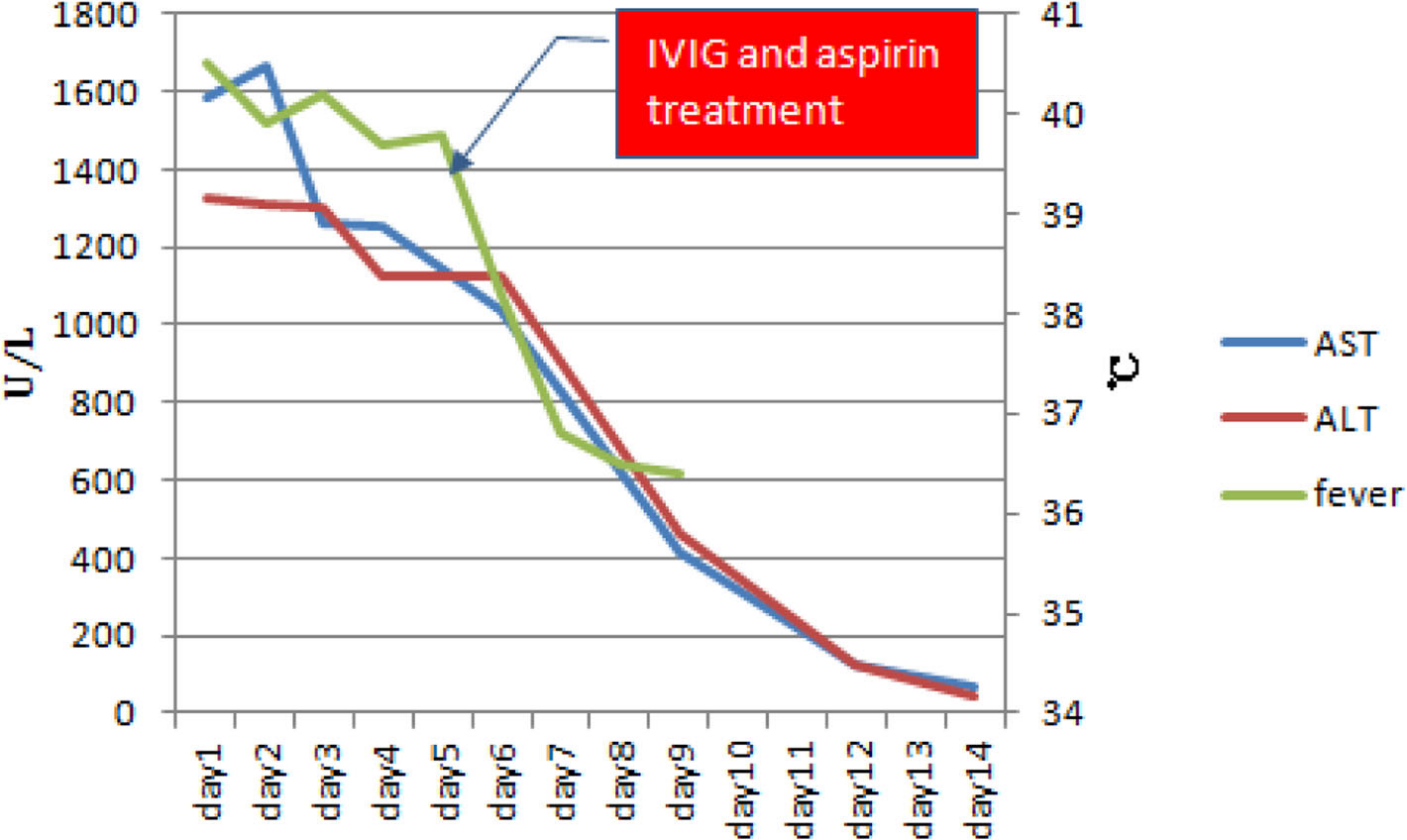
Trends of transaminases and temperature of the patient during hospitalization

**Fig. 3:**
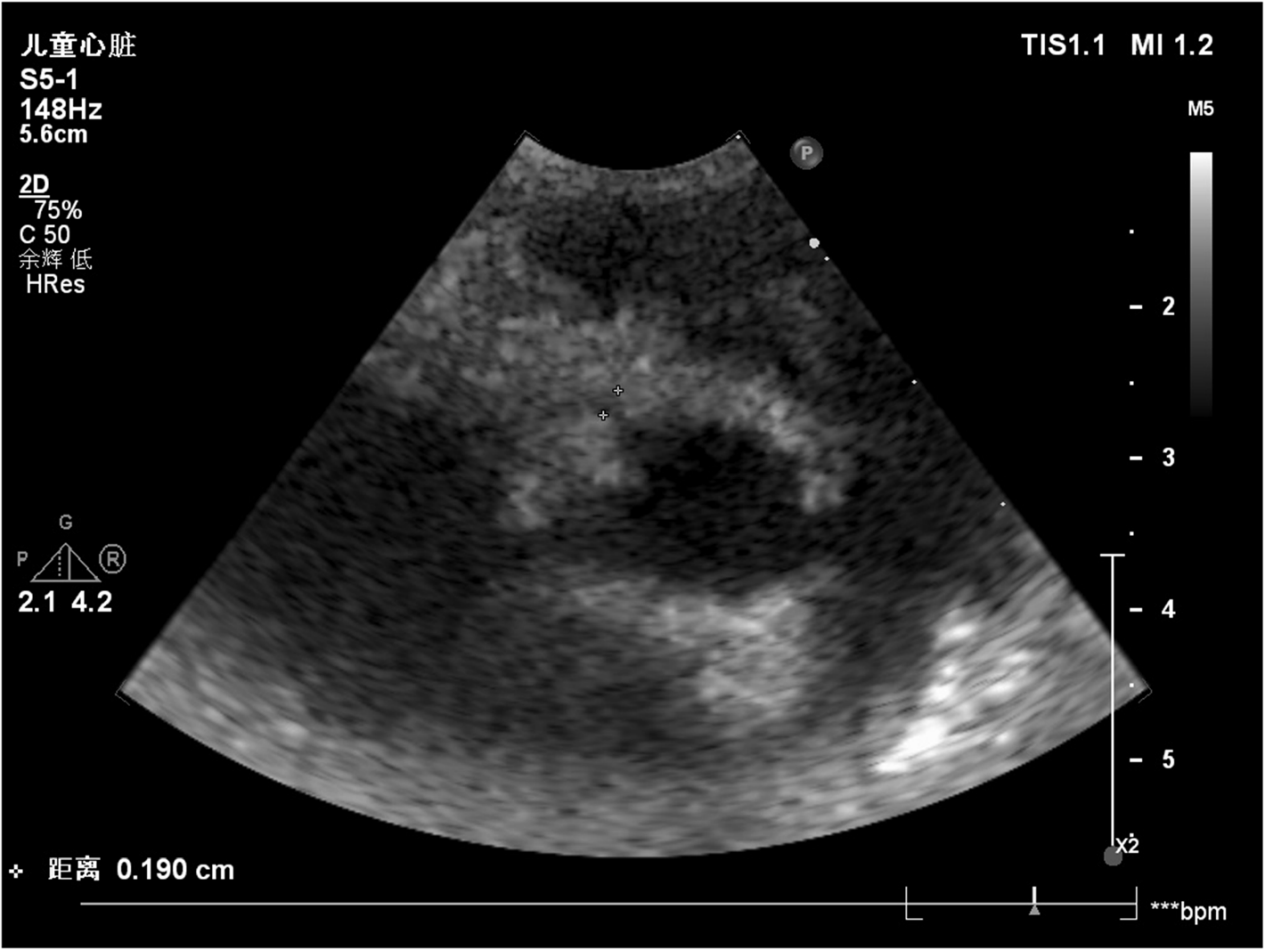
A gradual regression was observed in the CA aneurysm on echocardiography after 1 month

A 1-year-old Han nationality boy was admitted to the Department of Pediatrics, Shaoxing Keqiao Women and Children’s Hospital, Shaoxing, China, due to complaints of fever and cough occasionally for 2 days in March 2019. However, he did not experience breathing difficulty, chills, vomiting, diarrhea, yellowing of skin, convulsions, and rash. Also, the patient had no history of medication except for two 3-mL ibuprofen suspensions. He had always been in good health, and no abnormal findings were detected at birth and in family history. Laboratory findings were as follows: blood routine examination: White blood cell count (WBC): 8.5 (4–10) × 10^9^/L, percentage of neutrophils (N%): 65.5 (40–75), percentage of lymphocyte (L%): 22.8 (40–60), hemoglobin (Hb): 100 (110–150) g/L, platelets (PLT): 240 (100–300) × 10^9^/L), C-reactive protein (CRP): 130.9 (0–8) mg/L. The other test results were as follows: erythrocyte sedimentation rate (ESR): 55 (0–15) mm/h, serum alanine aminotransferase (ALT): 1327 (9–50) U/L, aspartate aminotransferase (AST): 1584 (15–40) U/L, total bilirubin: 7.81 (3.42–20.5) μmol/L, direct bilirubin: 2.60 (0.0–6.84) μmol/L, gamma glutamyl transferase (GGT): 248.9 (10.0–60.0) U/L, and albumin 41.8 (38.0–55.0) g/L. Urinalysis revealed the presence of pyuria. The nitrite test was negative. Serological tests for hepatitis A, B, C, D, E, F, Epstein-Barr virus (EBV), cytomegalovirus, and viruses in the respiratory tract were negative for acute infections. Abdominal ultrasonography showed normal liver, gallbladder, bile ducts, and pancreas. Chest radiographs suggested normal. Ceftriaxone (80 mg/kg.d, qd) was intravenously injected to intervene the possible lung or urinary tract infection. Compound glycyrrhizin injection 20 mL (2 mL/kg, qd) and reduced glutathione 0.3 g (30 mg/kg, qd) were administered to protect the liver. On day 4 post-hospitalization, a large red rash appeared on the chest and back of the child. The results of blood, urine, and throat cultures were negative, respectively, but the fever persisted despite intravenous administration of ceftriaxone for 3 days. The common cause of severely elevated transaminase in children is a viral infection, such as hepatitis A, B, C, D, E, F, EBV, and cytomegalovirus. Since the results for the above tests were negative for the child, and CRP was abnormally elevated, these were deemed incompatible with virus infection and attributed to bacterial infections, such as acute purulent cholecystitis that can cause elevated transaminases. Nonetheless, the child did not show any relevant clinical symptoms. Abdominal ultrasonography was normal, and ceftriaxone had poor anti-infective treatment effect, which further did not support the presence of bacterial infection. Bilateral diffuse dilatation of the CA (2.6 mm left and 3.1 mm right), especially the right, was detected on echocardiography on day 5 post-hospitalization (Fig. 1). The Z-scores of the left and right main coronary artery (CA) were 2.13 and 4.13, respectively. According to Z-score classification [2], the patient had a small CA aneurysm. In summary, the child with fever ≥5 days, CRP 130.9 mg/L, ESR 55 mm/h, Hb 100 g/L, elevated ALT level, urine ≥10 WBC/hpf, CA aneurysm, fulfilled the diagnostic criteria of atypical KD [2]. Ectasia was frequently detected in the atypical KD than typical KD [3]. Thus, he was diagnosed as atypical KD and treated with aspirin (35 mg/kg/day from days 5–8 of hospitalization and 4.5 mg/kg/day from day 9 of hospitalization for the following 3 months) and Intravenous immunoglobulin (IVIG) (2 g/kg/day) for 10 h on day 5 of hospitalization. Ceftriaxone was stopped on day 5 of hospitalization. After 24 h of treatment, clinical and laboratory parameters improved rapidly with regression of fever and aminotransferase levels on day 6 post-hospitalization. The trends of temperature and elevated transaminases are shown in Fig. 2. The child was discharged on day 9 of hospitalization. Also, a gradual regression was observed in the coronary blood vessels when normalized to the echocardiographic findings after 1 month (Fig. 3). Moreover, 2-months, 6-months, and 1-year follow-up did not show any recurrence of fever or an additional increase in the CA diameters corresponding to the maintenance dose (4.5 mg/kg/day for 3 months) of aspirin.

## Discussion

According to literature, elevated serum transaminases or gamma glutamyl transpeptidase is observed in 40–60% KD patients [4]. The majority of the patients show only a mild increase in transaminases, which is < 2-fold of the upper limit of normal, and only a few patients showed 10-fold of the upper limit of the normal (Table 1) [9]. Although severely elevated transaminase is rare, it could mislead the diagnosis and delay the treatment. Reportedly, high ALT and GGT values in the acute phase are related to IVIG resistance [4]. However, this phenomenon was not observed in our case. The main purpose of this case report was to acquaint the pediatricians that atypical KD may have masqueraded in various guises, and hence, the variability of this disease should not be ignored. To minimize the diagnostic delay, we investigated the clinical manifestations of atypical KD (Table 1).

**Table 1:** Case reports of atypical KD

Case source	Gender	Age (year)	Clinical manifestations	AST	ALT	Treatment	Prognosis
Turkey [5]	boy	6	fever, abdominal pain, jaundice, desquamation of fingers, bilateral conjunctival erythema	29 U/L	42 U/L	IVIG acetylsalicylic acid (ASA)	recovery
Turkey [5]	boy	2.5	fever, swelling, redness of upper surface of foot, cholestasis, and bilateral conjunctival erythema	44 U/L	51 U/L	IVIG ASA	good general health
Iran [6]	boy	1.3	fever, bilateral bulbar, non-purulent conjunctivitis, unilateral cervical lymphadenopathy, erythematous oral and pharyngeal mucosa	351 U/L	40 U/L	IVIG aspirin	good general health
New York [7]	boy	5	fever, emesis, right upper quadrant, abdominal pain, scleral icterus, bilateral nonexudative conjunctivitis, and inguinal rash with pealing	1.25 μkat/L	3.31 μkat/L	IVIG aspirin	recovery
New York [7]	girl	4	fever, Jaundice, abdominal pain, itchy rash on the arms and legs, maculopapular rash red, cracked lips, a red tongue, and peeling skin in the groin and on the hands	0.9 μkat/L	1.35μkat/L	IVIG aspirin	recovery
New York [8]	boy	2.5	fever, bilateral non-suppurative conjunctivitis, right upper quadrant tenderness, gallbladder hydrops persistent, abnormal liver function tests	115 U/L	146 U/L	IVIG ursodeoxycholic acid, intravenous antibiotics	good general health
China	boy	1	fever, rash, bilateral diffuse dilatation of CA	1584 U/L	1327 U/L	IVIG aspirin	recovery

Since the cause of KD was unknown, it was speculated to be associated with the region, year, gender, season, family history, and genetics. Accumulating evidence linked KD to tropospheric wind patterns, which suggested that the transport of an agent when inhaled by genetically susceptible children, triggers the immunological cascade of KD [2]. In addition to CA abnormalities, hepatic dysfunction is also a common complication during the acute KD episode. Reportedly, 90.95% of KD patients have at least 1 abnormal liver function test, wherein hypoalbuminemia is the most prevalent type, followed by elevated AST, low total protein, low albumin/globulin ratio, and hyperbilirubinemia; however, the contributing factors are yet unclear and could be associated with inflammatory mediators, infectious agents, therapy, or a combination of the above [10]. Fei et al. reported continuous veno-venous hemodiafiltration that could rapidly reduce the levels of interleukin-6 (IL-6) and tumor necrosis factor-alpha (TNF-α) and improve the organ function of KD complicated with multiple organ dysfunction syndromes [11]. This concept provided a rescue therapy in children with KD complicated with severe organ damage. Intriguingly, Sundel et al. suggested a process initiated by an innate immune response to explain the pathophysiology of KD. It involved a reaction mediated by the acquired immune system, resulting in the loss of blood vessel structural integrity, arterial wall dilatation, and aneurysm formation [12]. Fei and Sundel proposed that the immune response was involved in the pathogenesis of KD [11, 12]. Severely elevated transaminases of atypical KD might be related to severe immune damage to the liver.

Hua et al. reported that in patients ≤6-months-old, total fever duration of ≥8 days, delayed diagnosis, and albumin (ALB) ≤35.9 g/L were independent risk factors for acute and subacute KD combined with CA lesions (CAL) [13]. Shi et al. reported that delayed hospitalization is one of the factors of the increased risk of CAL in patients with atypical KD [14]. Yunjia et al. demonstrated that patients aged ≤1-year-old receive IVIG treatment after day 10 of illness, and IVIG non-responders were associated with the regression in persistent CA aneurysms (CAA) [15]. In summary, timely admission to the hospital and prompt treatment for KD patients is essential. Therefore, early recognition of the clinical symptoms of atypical KD, such as severe liver damage, is vital for the treatment of the disease.

## Conclusion

KD could lead to severe complications, such as CAAs and thromboembolic occlusions. Thus, early diagnosis of the disease is an urgent requirement. Atypical KD clinical symptoms are diverse. Severely elevated transaminases could be one of the manifestations of atypical KD.

## Data Availability

All data generated or analyzed during this study are included in the article.

## Abbreviations

KD: Kawasaki disease
CA: Coronary artery
CRP: C-reactive protein
IVIG: Intravenous immunoglobulin
WBC: White blood cell count
N%: Percentage of neutrophils
L%: Percentage of lymphocyte
Hb: Hemoglobin
PLT: Platelets
ESR: Erythrocyte sedimentation rate
ALT: Serum alanine aminotransferase
AST: Aspartate aminotransferase
GGT: Gamma glutamyl transferase
EBV: Epstein-Barr virus
IL-6: Interleukin-6
TNF-α: Tumor necrosis factor-alpha
ALB: Albumin
CAL: Coronary artery lesions
CAA: Coronary artery aneurysms
